# Enhancement of Vascularization and Ovarian Follicle Survival Using Stem Cells in Cryopreserved Ovarian Tissue Transplantation—A Systematic Review

**DOI:** 10.3390/biology13050342

**Published:** 2024-05-14

**Authors:** Luca Zaninović, Marko Bašković, Davor Ježek, Dubravko Habek, Zenon Pogorelić, Ana Katušić Bojanac, Vesna Elveđi Gašparović, Lana Škrgatić

**Affiliations:** 1 Scientific Centre of Excellence for Reproductive and Regenerative Medicine, School of Medicine, University of Zagreb, Šalata 3, 10 000 Zagreb, Croatia; 2 Department of Obstetrics and Gynecology, University Hospital Centre Zagreb, Petrova ulica 13, 10 000 Zagreb, Croatia; 3 School of Medicine, University of Zagreb, Šalata 3, 10 000 Zagreb, Croatia; 4 Department of Pediatric Surgery, Children’s Hospital Zagreb, Ulica Vjekoslava Klaića 16, 10 000 Zagreb, Croatia; 5 Croatian Academy of Medical Sciences, Kaptol 15, 10 000 Zagreb, Croatia; 6 Department of Histology and Embryology, School of Medicine, University of Zagreb, Šalata 3, 10 000 Zagreb, Croatia; 7 Department of Transfusion Medicine and Transplantation Biology, University Hospital Centre Zagreb, Kišpatićeva ulica 12, 10 000 Zagreb, Croatia; 8 Department of Obstetrics and Gynecology, Clinical Hospital Merkur, Zajčeva ulica 19, 10 000 Zagreb, Croatia; 9 School of Medicine, Catholic University of Croatia, Ilica 242, 10 000 Zagreb, Croatia; 10 Department of Pediatric Surgery, University Hospital of Split, Spinčićeva ulica 1, 21 000 Split, Croatia; zpogorelic@kbsplit.hr; 11 School of Medicine, University of Split, Šoltanska ulica 2a, 21 000 Split, Croatia; 12 Department of Medical Biology, School of Medicine, University of Zagreb, Šalata 3, 10 000 Zagreb, Croatia

**Keywords:** ovary, ovarian tissue, cryopreservation, transplantation, stem cell, angiogenesis, oxygenation, follicle survival

## Abstract

**Simple Summary:**

Increases in survival rates from malignant diseases has introduced the need to ensure fertility preservation treatment for cancer patients. Cryopreservation of ovarian tissue is a relatively new technology in which fragments of ovarian tissue are sampled and cryopreserved in order to be re-transplanted into the patient after completion of treatment. The main shortcoming of this method is tissue re-transplantation without the formation of vascular anastomoses, which limits the blood flow and oxygen supply to the transplanted tissue until a novel vascular network is established. In this systematic review, we have gathered reports that study how co-transplantation of mesenchymal cells together with thawed ovarian tissue contributes to the process of revascularization and, consequently, the preservation of follicles and ovarian tissue. The conclusion that stem cells contribute to ovarian tissue preservation was drawn from histological analysis of ovarian tissue, the detection of factors that contribute to vascular formation and analysis of sex hormone levels which indirectly indicate the resumption of ovarian function.

**Abstract:**

The increase in cancer survival rates has put a focus on ensuring fertility preservation procedures for cancer patients. Ovarian tissue cryopreservation presents the only option for prepubertal girls and patients who require immediate start of treatment and, therefore, cannot undergo controlled ovarian stimulation. We aimed to provide an assessment of stem cells’ impact on cryopreserved ovarian tissue grafts in regard to the expression of growth factors, angiogenesis promotion, tissue oxygenation, ovarian follicle survival and restoration of endocrine function. For this systematic review, we searched the Scopus and PubMed databases and included reports of trials using murine and/or human cryopreserved ovarian tissue for transplantation or in vitro culture in combination with mesenchymal stem cell administration to the grafting site. Of the 1201 articles identified, 10 met the criteria. The application of stem cells to the grafting site has been proven to support vascular promotion and thereby shorten the period of tissue hypoxia, which is reflected in the increased number of remaining viable follicles and faster recovery of ovarian endocrine function. Further research is needed before implementing the use of stem cells in OT cryopreservation and transplantation procedures in clinical practice. Complex ethical dilemmas make this process more difficult.

## 1. Introduction

Advancements in the diagnosis and treatment of malignant diseases have led to an increase in patient survival rates and put the focus on ensuring an adequate quality of life after recovery. Cancer, in addition to an increasing number of non-oncological systemic diseases, requires invasive treatment with chemotherapy, radiotherapy, as well as bone marrow transplantation, which can cause premature depletion of the ovarian follicle reserve. Therefore, fertility preservation options should be considered in individuals of reproductive age or younger before receiving gonadotoxic therapy [1,2,3]. While mature oocyte and embryo cryopreservation present standard-of-care approaches, ovarian tissue cryopreservation remains the only option for prepubertal girls and patients who require immediate start of treatment (and hence cannot undergo controlled ovarian stimulation) [4,5]. Moreover, transplanted ovarian tissue (OT) offers the possibility of restoring not only fertility but also endocrine ovarian function [6]. The most significant limiting factor of ovarian tissue survival after transplantation is the period of tissue ischemia while awaiting for sufficient vascularization to be established. Observed loss of primordial follicles is over 50% in the first 4 to 5 days following grafting [2,5,7]. A reduction in the post-transplantation period of hypoxia is hypothesized to improve ovarian graft efficiency. Local administration of angiogenic factors, such as sphingosine-1-phosphate, vascular endothelial growth factor and angiopoietin, to the graft site has demonstrated advancements in angiogenesis and reperfusion restoration [8,9,10]. A novelty in cryopreserved ovarian tissue transplantation involves stem cells capable of indefinite self-renewal and the production of daughter cells that differentiate depending on the cellular milieu. Their addition to the ovarian tissue graft has demonstrated positive effects on angiogenesis, tissue oxygenation and follicle survival [11,12]. Only one review article on this specific topic pools results from five studies and offers an overview of stem cell utilization in ovarian tissue cryopreservation and transplantation [12].

This systematic review aims to provide a detailed assessment of stem cells’ impact on cryopreserved ovarian tissue grafts in regard to the expression of growth factors, angiogenesis promotion, tissue oxygenation, ovarian follicle survival and restoration of endocrine function.

## 2. Materials and Methods

### 2.1. Study Design and Search Strategy

The study was performed according to the Preferred Reporting Items for Systematic Reviews and Meta-Analysis (PRISMA) statement.

On 10 October 2023, PubMed and Scopus databases were searched. The following search criteria were applied: ((ovari*) OR (ovari* tissue) OR (ovari* cortex)) AND ((cryopreserv*) OR (bank*) OR (vitrificat*) OR (freez*)) AND ((stem cell*) OR (mesenchymal stem cell*) OR (MSC) OR (umbilical cord mesenchymal stem cell*) OR (UC-MSC*) OR (human umbilical cord mesenchymal stem cell*) OR (hUC-MSC*) OR (adipos*) OR (endothel*) OR (bone marrow)). No search filters or text analysis tools were used, and all articles from the moment of the foundation of the database until the day the search was performed were evaluated.

### 2.2. Eligibility Criteria

We included randomized controlled trials using murine and/or human cryopreserved ovarian tissue for transplantation or in vitro culture in combination with mesenchymal stem cell administration to the grafting site. We considered outcomes that would allow us to indirectly assess the impact of post-transplant period ischemia, which appears to be the critical factor for ovarian tissue graft survival. Study results undergoing systematization and qualitative evaluation included histological analysis of ovarian tissue, immunohistochemistry assays providing information about angiogenesis promotion and follicle growth, gene expression analysis by polymerase chain reaction (PCR) and Western blotting, as well as ovarian endocrine function indicators. Studies investigating the effect of oogonial or embryonic stem cells were excluded in addition to the ones in which isolated cryopreserved follicles were transplanted separately or through artificial ovary creation. The systematic review protocol was registered with the International Prospective Register of Systematic Reviews (PROSPERO, ID 524261).

### 2.3. Screening Process, Critical Appraisal and Data Collection Process

**Figure 1:**
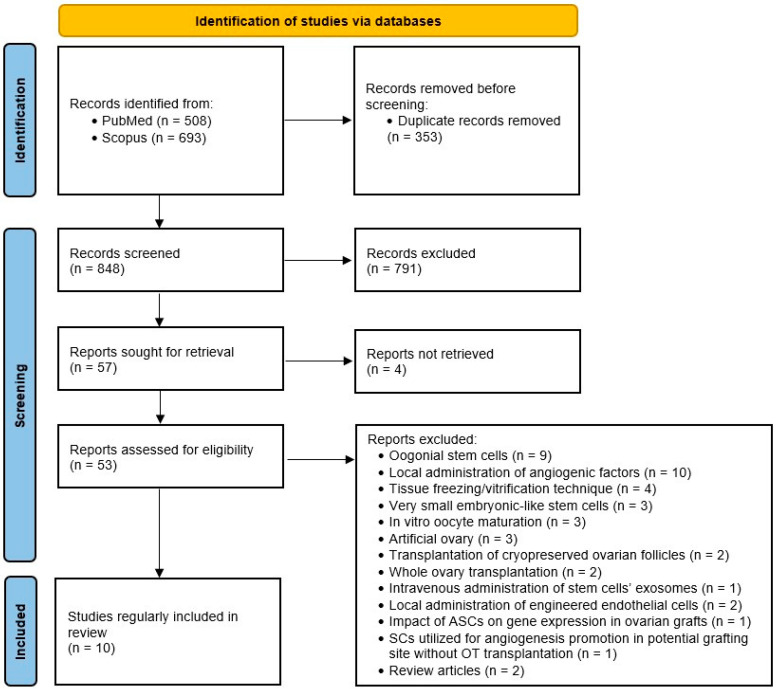
PRISMA flow diagram.

Following duplicate removal, studies were selected through a two-stage process. Firstly, two authors independently assessed the eligibility of study records by title and abstract screening. In cases of disagreement, the studies in question were directly intended for full-text screening. In the next step, the same two researchers screened full-text articles for inclusion and discussed inconsistencies until a consensus was obtained (see flow diagram in Figure 1. summarizing study selection process).

The data extraction form was designed by the consensus of all the authors. A detailed list of sought-after information is stated in Table S1. The independent review author retrieved data from eligible studies.

## 3. Results

### 3.1. Search Results

We obtained 1201 records in total from database searching. After duplicate removal, we screened 848 records by titles and abstracts, and 791 of them were excluded for being unsuitable. We managed to retrieve 53 full-text documents for further evaluation. Ten studies met the criteria and were included in the review. A summary of the characteristics of the included studies is available in Table 1. During the critical appraisal process, data from another study seemed to possibly contribute to the informativeness of the review; however, we decided not to include it, as the authors did not use the ovarian tissue but solely utilized adipose tissue stem cells (ASCs) for angiogenesis promotion in potential grafting sites [13].

### 3.2. Experimental Design of Included Studies

Three studies investigated the early development of in vitro ovarian culture with the addition of mesenchymal stem cells [14,15,16]. Two of these studies utilized human ovarian tissue obtained from female donors undergoing therapeutic surgical operations for reasons such as endometriosis or teratoma [14,15]. The obtained tissue samples were then dissected into fragments of just a few millimeters in diameter and then vitrified. The third in vitro study used ovarian cortical tissue from BALB/c mice. Stem cells were either isolated from connective tissue and donated human umbilical cords or bone marrow stem cells that were commercially acquired. Examples of 3D in vitro cultures were formed. Thawed ovarian cortical tissue encapsulated with Matrigel^®^ (BD, Franklin Lakes, NJ, USA) was placed in transwell chambers that were inserted into a well plate pre-seeded with stem cells. Due to the permeability of the polycarbonate membrane, the cytokines and growth factors secreted by stem cells were able to migrate to the upper chambers and affect ovarian tissue development [14,16]. Hosseini et al. directly transferred thawed OT to the upper chamber of the transwell plate filled with serum-free culture medium [15].

The group of investigators, led by Lamarão Damous, published three consecutive studies on Wistar rats [17,18,19]. After bilateral oophorectomy, whole ovaries were cryopreserved by a slow-freezing procedure. Each animal received whole-thawed ovary autologous transplantation without vascular anastomosis in the retroperitoneum. Adipose tissue stem cells were isolated from inguinal subcutaneous tissue from 10-week-old Wistar rats. The animals were then randomized into experimental groups according to transplantation of ovaries alone, application of vehicle in addition to the ovarian tissue, or co-transplantation with ASCs. After publishing two studies utilizing whole stem cells, either injected directly or transplanted in extracellular matrix bioscaffold, in their latest study, they introduced a novel cell-free approach where they injected ovarian grafts with the secretome isolated from the ASC culture [18].

Four studies obtained donated human ovarian tissue for their experiments. Female donors aged 20–35 years underwent oophorectomy for non-ovarian pathology. After being cryopreserved by a slow-freezing procedure and subsequently thawed, ovarian tissue fragments were transplanted to SCID or BALB/c mice. Grafting sites differed between studies, ranging from orthotopic intraperitoneal transplantation to heterotopic graft positioning under the renal capsule, subcutaneously, or into the back muscle region [20,21,22,23]. Two studies used commercially acquired human ASCs and performed a two-step procedure. Firstly, the anticipated grafting site was prepared with ASC-loaded fibrin implants 14 days before OT grafting. Ovarian tissue fragments were then placed below the remaining fibrin layer [22,23]. The other two studies isolated human mesenchymal stem cells from donated umbilical cord and bone marrow tissues. Xia et al. divided their laboratory mice into four groups depending on whether they were grafted with human OT alone, OT in combination with the vehicle, growth factors, or human bone marrow mesenchymal stem cells. Ovarian fragments were xenotransplanted subcutaneously with the cortical side adhering to the skin and the medullar side adhering to the fascia [21]. Cheng et al. took it a step further and reviewed the fact that an SC’s secretome varies significantly depending on the cellular surroundings. They preconditioned their human umbilical cord mesenchymal stem cells (hUC-MSCs), predicting that a hypoxic environment will enhance proangiogenic factor secretion. The hUC-MSCs were placed in a hypoxic incubator with only 3% O_2_ for 48 h before transplantation [20].

### 3.3. Histological Evaluation of Transplanted Ovarian Tissue

Ovarian tissue samples intended for histological evaluation were fixed in 4% formaldehyde and embedded in paraffin. Paraffin blocks were then serially cut into 5 μm sections and stained by hematoxylin and eosin.

In vitro studies evaluated the impact of co-culturing of thawed ovarian tissue with SCs on the survival of different stages of ovarian follicles [14,15,16]. Ovarian tissue analysis demonstrated that, after thawing, 87.34% of follicles preserved normal histological morphology. The overall number of viable follicles slightly declined during the culturing period without a statistically significant difference between monoculture and co-culture groups. On day two, the number of primordial follicles decreased and the number of primary follicles increased in both the co-cultured and control groups in comparison to non-cultured ovarian tissue on day 0 (*p* < 0.05). Up until culturing day 8, the proportion of primordial follicles was significantly (*p* < 0.05) lower in a group of OT co-cultured with 3 × 10^5^ hBM-MSCs than in the monoculture control group [15]. Xu et al. showed no statistically significant difference in the total number of follicles between the co-cultured and control group, However, co-culturing with Matrigel^®^ and hUC-MSCs was proven to increase the number of morphologically normal follicles and reduce the level of follicular atresia. In addition, the percentage of primordial, primary and secondary follicles in SC co-cultured groups was higher in comparison to the control monoculture group [16]. In the study by Jia et al., there was no significant difference in the total number of follicles between the group co-cultured with SCs and the serum-free culture medium group. Nevertheless, during the first four days of culturing, the percentage of primordial follicles was higher in the SCs group than in the SFCM group. After day 4, no statistically significant difference between groups was observed [14].

In studies performed on Wistar rats, follicular viability measured by trypan blue staining showed no significant difference between groups [17,19]. In the study where OT and SCs were co-cultured without vehicle application, the predominance of primordial and preantral over mature follicles was pronounced in both groups [19]. The other two studies showed various stages of follicle maturation, ranging from primordial to postovulatory corpora lutea and albicans, in the vehicle-treated group. The ovarian grafts treated with SCs or their secretome exhibited diffuse fibrosis with blood vessel wall hyalinization and lumen obliteration accompanied by follicular atrophy and foreign body granuloma formation around sutures [17,18].

A study by Cheng et al. measured follicle density (number of follicles/graft volume [mm^3^]) in human ovarian grafts. It was notably higher when hUC-MSCs and OT were co-transplanted to mice in comparison to OT alone. In addition to total follicle density, the density of resting follicles was significantly increased and the rate of atresia decreased in the SCs group [20]. Xia and colleagues’ findings were consistent with the abovementioned evidence even though they did not calculate density but rather exhibited their results as the total number of follicles in a 400-fold microscopy field. In addition, they histologically estimated the microvascular density of grafts. Over time, microvascular formation increased in all four groups. Nevertheless, the density was persistently higher in the SCs+OT group. There was no statistically significant difference between the other three groups [21].

### 3.4. Immunohistochemistry

Immunohistochemistry staining was used to detect the presence of specific protein markers. Previous studies have demonstrated that CD34 is expressed in endothelial cell filopodia at sites of active angiogenesis. It acts as an anti-adhesive molecule during lumen formation and prevents the adhesion of contralateral apical endothelial cell surfaces [24]. CD31 (PECAM-1; platelet/endothelial cell adhesion molecule-1) is a transmembrane protein that potentiates adhesion between adjacent endothelial cells [25]. The immunohistochemical detection of these two protein markers has been used in angiogenesis and microvessel density assessment.

In vitro studies showed a general time-dependent increase in microvessel density over the first 5 days of culturing. The microvessel density (MVD) calculated by CD34 expression was higher in the hUC-MSC group [14]. Furthermore, the addition of Matrigel^®^ to hUC-MSC and OT 3D culture promoted vascular formation, but the difference in CD31 expression between groups was not statistically significant [16].

According to CD31 staining, MVD of xenograft human ovarian tissue with hypoxic-preconditioned hUC-MSCs was significantly higher than that of the normoxic-treated group on day 3, but the difference diminished by day 7. There was no statistically significant difference in CD34 expression between hypoxic and normoxic-preconditioned hUC-MSC groups; however, the expression was higher in both of them compared to the OT group [20]. Caccittola et al. demonstrated that, when the transplantation site was prepared with SCs implanted 14 days before grafting, murine vessels with CD34 positive expression were found earlier than in the non-prepared group (day 3 vs. day 10). The endothelial area of murine vessels significantly increased from day 3 to days 10 and 21 in the OT group, while stationary findings were observed in the 2-step group. The statistically significant difference in total endothelial area (human + murine) diminished by day 10. No significant difference in human MVD was observed at any time point [23]. In contrast, Manavella et al. observed that human MVD characterized by CD34 expression was significantly higher in the ASC preconditioned group. They also proved the differentiation of ASCs into CD34-positive endothelial cell lineage by co-expression of both CD34 and eGFP antigens. These differentiated cells were detected in 12.15% of human blood vessels in ovarian tissue grafts [22].

Caspase-3 (cysteine-dependent aspartate-specific protease-3) is a member of the family of enzymes accountable for the induction and execution of programmed cell death through the process of apoptosis. Active caspase-3 presents a cleaved form of the enzyme that initiates an apoptotic signaling cascade. AC-3 immunohistochemical staining was used as an indicator of cell death induction [26,27].

The number of AC-3 positive follicles was significantly higher in vitro culture where only serum-free culture medium was added to OT in contrast to the group co-cultured with hUC-MSCs (51.0% vs. 11.75% on day 5, *p* = 0.019) [14]. In the case of human ovarian tissue grafted to BALB/c mice, a significant reduction in AC-3 positive primordial follicles was observed in the MSC group compared to the other groups on day 7 [21]. These results demonstrate that co-culturing of ovarian cortex tissue with stem cells could notably inhibit post-transplantational follicle apoptosis. In contrast, Lamarão Damous et al. exhibited how co-transplantation of ASCs and OT in Wistar rats promoted apoptosis measured by AC-3 staining (2.51 vs. 3.11 in ASC group, *p* < 0.05) [17]. In studies where lower rates of follicular apoptosis was observed, mesenchymal stem cells were transplanted into the extracellular matrix bioscaffold (Matrigel^®^). The direct injection of ASCs may have overstimulated inflammatory response and consequently caused fibrosis and increased apoptosis rates in experiment on Wistar rats.

Immunohistochemical staining of the cellular proliferation marker Ki-67 was used to evaluate the growth of primordial follicles in vitro. Ki-67 is a non-histone DNA-binding protein that is upregulated during cell mitosis [28]. The primordial follicles were considered growing when at least one granulosa cell was Ki-67 positive. There was no significant difference between follicle proliferation index in thawed ovarian tissue co-transplanted with hypoxic or normoxic-pretreated SCs [20].

Vascular endothelial growth factor (VEGF) is an important growth factor in angiogenesis. It holds potential as a predictive marker of neovascularization in grafted OT. Manavella et al. observed significantly higher VEGF-positive staining concentrations in a group where human OT was grafted to SCID mice with the addition of ASCs [22].

Cacciottola et al. assessed the tissue oxygenation by IHC through measuring the expression of oxidative stress markers: hypoxia-inducible factor 1a (HIF1a), nuclear factor erythroid 2-related factor 2 (Nrf2) and 8-hydroxydeoxyguanosine (80HdG). Lower values of HIF1a positive staining primordial follicles were observed in a 2-step (ASC+OT) group 3 days after transplantation. No significant difference in HIF1a expression was found in stroma cells between the two groups. Nrf2 and 80HdG were not expressed either in granulosa cells nor oocytes, regardless of the group. At the same time, greater values of the abovementioned oxidative stress markers were observed more in grafted than in non-grafted groups, with no significant difference between the OT and ASC+OT groups [23].

### 3.5. TUNEL Staining

TUNEL (terminal deoxynucleotidyl transferase dUTP nick end labeling) assay was used to evaluate the ratio of apoptotic cells in ovarian tissue grafts. Terminal deoxynucleotidyl transferase (TdT) is a DNA polymerase that attaches nucleotides to the ends of DNA fragments produced by endonuclease enzymes. The action of TdT is limited to 3′ phosphate groups that are specific for the process of apoptosis but not necrosis [29]. TdT was used to attach previously fluorescently labeled dUTP to the exposed 3′ ends of DNA fragments and thereby mark apoptotic cells, allowing for their detection by fluorescence microscopy. In vitro studies showed that the ratio of apoptotic cells was significantly lower in the co-culture group (OT+hBMSCs) compared to the monoculture group at all time points [15,16].

The findings by Lamarão Damous and colleagues were inconsistent. In their first study from 2015, co-culturing with ASCs increased cell apoptosis rates as measured by both AC-3 and TUNEL assay (vehicle 0.17 vs. ASCs 0.52, *p* < 0.05) [17]. When they added only the ASC secretome instead of the whole stem cells to the ovarian tissue graft, the percentage of viable follicles decreased and the apoptosis rates measured by TUNEL increased (vehicle 0.11 vs. secretome 1.72, *p* < 0.05). In contrast, immunolabeling by AC-3 demonstrated no difference in apoptosis levels between groups [18]. In their final experiment on Wistar rats, co-culturing did not result in any statistically significant difference in performed assays between groups [19]. Administration methods of stem cells may affect the dose delivered and the tissue response. Tissue density and counterpressure must be taken into consideration. Direct injection of stem cells may cause abrupt dismissal of foreign antigens and induce an inflammatory response. Delivery of SCs through an acellular bioscaffold enables the gradual and continuous release of proangiogenic factors. It also prevents cells from migrating to other sites and reduces inflammatory response. Alternative to cellular techniques is an addition of a stem cell secretome that contains angiogenic and antiapoptotic growth factors [30,31,32].

The percentages of apoptotic stroma cells and follicles were separately estimated in human ovarian tissue co-transplanted to SCID mice along with normoxic or hypoxic-treated hUC-MSCs. The rate of TUNEL-positive stromal cells and follicles was significantly higher in the OT group as well as the normoxic-treated group compared to non-grafted controls. Notably, a lower percentage of both—TUNEL positive stromal cells and apoptotic follicles were detected in the H-MSCs+OT group compared to the N-MSCs+OT and OT group (*p* = 0.004, *p* < 0.001) in the post-transplantation day [20]. These findings indicate that the addition of SCs to thawed human ovarian tissue grafts could suppress cell apoptosis rates.

### 3.6. RT-PCR and Western Blot

More discrete changes in the expression of growth and transcriptional factors are detectable by RT-PCR analysis and Western blotting. Xu et al. in their study observed how the addition of hUC-MSCs to in vitro OT culture increased the mRNA expression of gene coding for angiogenesis-related growth factors (VEGFA, ANGPT2, and IGF1). Furthermore, the expression of glycogen synthase kinase-3 beta was downregulated, whereas the upregulation in β-catenin expression was observed indicating Wnt/β-catenin signaling pathway activation accountable for angiogenesis promotion [16].

The results of in vivo studies using human ovarian tissue were consistent with these observations. Relative quantification by PCR determined significantly higher expression of VEGF mRNA in a group where OT was co-transplanted with ASCs as well as in the cases when the transplantation site was prepared with ASC-loaded fibrin implants 14 days before OT transplantation [22,23]. After 21 days, the difference in mRNA expression levels diminished [23]. Western blotting also showed significantly higher VEGFA expression in the H-MSCs+OT group than the N-MSCs+OT group, while both groups had higher VEGFA levels than the OT group. The same was true for HIF1 expression [20]. Analysis of FGF2 expression demonstrated contrary results. While Xia et al. described higher expression of this growth factor in the SCs group, in the study by Manavella et al., the difference between groups was not statistically significant [21,22]. The expression of the Ki67 proliferation marker was higher in the co-culture group [21]. Preconditioning of stem cells in a hypoxic environment results in expression of oxidative stress markers and activation of signaling and metabolic pathways that help cells adapt to unfavorable conditions. HIF1 as a DNA-binding transcription factor indirectly enhances vascularization by activation of a series of genes in response to hypoxic conditions [33,34]. The upregulation of angiogenesis factors such as VEGFA is protective against SCs and primordial follicle apoptosis [35,36]. Ki67 staining indicates activation of flat granulosa cells and follicular growth. Higer expression of this cycle-associated nucleoprotein suggests follicular activation as the major cause of follicle pool depletion [37].

### 3.7. Hormonal Status

An alternative method for the transplanted ovarian tissue function assessment is the measurement of the concentration of sex hormones normally secreted by ovaries and the pituitary gland.

In an in vitro study by Hosseini et al., the concentration of 17-beta-estradiol (E2) measured by an ELISA kit revealed higher concentrations in the co-culture group compared to the OT group on day 4 [15]. Co-transplantation of H-MSCs with human OT caused increased secretion of anti-Mullerian hormone compared to N-MSCs+OT and OT groups. By day 7, statistically significant differences in hormone levels between groups diminished while the concentration in all three groups increased. Similar changes in concentration levels were observed for E2 and progesterone, except for the N-MSCs+OT group where the E2 level on day 7 was not significantly higher than on day 3. On day 7, the progesterone level in H-MSCs+OT was not significantly higher than in the N-MSCs+OT group. On the contrary, the concentration of follicle-stimulating hormone in groups co-transplanted with preconditioned SCs was significantly lower than in the monoculture group [20].

In the studies on Wistar rats, all animals resumed the estrous cycle. In the study from 2015, the animals co-treated with ASCs showed earlier cycle resumption compared to the control group, which was not the case in the following experiment when both groups of animals treated with either vehicle or ASC secretome resumed the estrous cycle on the same day [17,18,19].

**Table 1:** Studies included in the systematic review.

Study	Type of Study	Origin of Ovarian Tissue Grafts	Age of Human Donors	Method of Cryopreservation	Ovarian Tissue Fragment Size	Transplantation Site	Experimental Groups	Type of Stem Cells	The Acquisition of SCs
Jia et al., 2017 [14]	in vitro	Human	29–37	vitrification	2 × 2 × 1–2 mm	in vitro serum-free culture medium	1. monoculture of OT 2. OT with hUC-MSCs 3. OT with SF cm	hUC-MSCs (before 6 passages)	3 human donors
Hosseini et al., 2020 [15]	in vitro	Human	18–42	vitrification	5 × 10 × 1 mm	in vitro serum-free culture medium	1. co-culture of OT and 3 × 10^5^ hBM-MSCs 2. monoculture of OT	hBM-MSCs (passages 4 and 5)	commercially acquired
Xu et al., 2022 [16]	in vitro	8-week-old BALB/c mice	N/A	vitrification	N/A	in vitro 3D culture system medium	1. OT without Matrigel^®^ or hUC-MSCs 2. OT with Matrigel^®^ 3. OT with hUC-MSCs 4. OT with Matrigel^®^ and hUC-MSCs	hUC-MSCs	human donors
Lamarão Damous et al., 2015 [17]	RCT in vivo	12-week-old Wistar rats	N/A	slow-freezing	whole ovary	retroperitoneally without vascular anastomosis to Wistar rats	1. OT with Gelfoam 2. OT with rASCs and Gelfoam	rASCs transgenic for green fluorescent protein (until passage 3)	10-week-old Wistar rat
Lamarão Damous et al., 2015 [19]	RCT in vivo	12-week-old Wistar rats	N/A	slow-freezing	whole ovary	retroperitoneally without vascular anastomosis to Wistar rats	1. OT with low glucose DMEM 2. OT with 5 × 10^4^ ASCs	rASCs transgenic for green fluorescent protein (until passage 3)	10-week-old Wistar rats
Lamarão Damous et al., 2018 [18]	RCT in vivo	12-week-old Wistar rats	N/A	slow-freezing	whole ovary	retroperitoneally without vascular anastomosis to Wistar rats	1. monoculture of OT 2. OT with vehicle 3. OT with ASCs secretome	secretome of rACSs (until passage 3)	10-week-old Wistar rats
Xia et al., 2015 [21]	RCT in vivo	Human	21	slow-freezing	2.5 × 2.5 × < 1 mm	subcutaneously to 33 8-week-old athymic BALB/c mice	1. monoculture of OT 2. OT with Matrigel^®^ 3. OT with Matrigel^®^ and FGF2 4. OT with Matrigel^®^ and hBM-MSCs	hBM-MSCs	five human donors (3 M, 2 F)
Manavella et al., 2019 [22]	RCT in vivo	Human	25–35	N/A	5 × 4 × 1 mm	intraperitoneally to 10 SCID mice	1. grafting site prepared with ASC-loaded fibrin implant 14 days before OT grafting 2. OT alone	ASCs transgenic for green fluorescent protein	commercially acquired
Cacciottola et al., 2021 [23]	RCT in vivo	Human	20–35	slow-freezing	6 × 4 × 1.5 mm	back muscle region of nude mice aged 8–15 weeks	1. grafting site prepared with 1.5 × 10^6^ ASC-loaded fibrin implant 14 days before OT grafting 2. OT alone	hASCs (passages 5 and 6)	commercially acquired from female donors
Cheng et al., 2022 [20]	RCT in vivo	Human	20–31	slow-freezing	round (diameter of 3 mm)	under the renal capsules of 64 8-week-old SCID mice	1. OT without hUC-MSCs 2. OT with 2 × 10^6^ normoxic-treated hUC-MSCs 3. OT with 2 × 10^6^ hypoxic-treated hUC-MSCs 4. bilateral oophorectomy without OT	hypoxia-preconditioned hUC-MSCs (between passages 5 and 8)	3 human donors

OT—ovarian tissue, hUC-MSCs—human umbilical cord mesenchymal stem cells, SFCM—serum-free culture media, hBM-MSCs—human bone marrow mesenchymal stem cells, BALB/c—Bagg albino, N/A—not applicable, RCT—randomized controlled trial, ASCs—adipose-derived stem cells, rASCs—rat ASCs, hASCs—human ASCs, DMEM—Dulbecco’s modified eagle medium, FGF2—fibroblast growth factor 2, M—male, F—female, SCID—severe combined immunodeficiency.

### 3.8. Risk of Bias in Primary Studies

**Figure 2:**
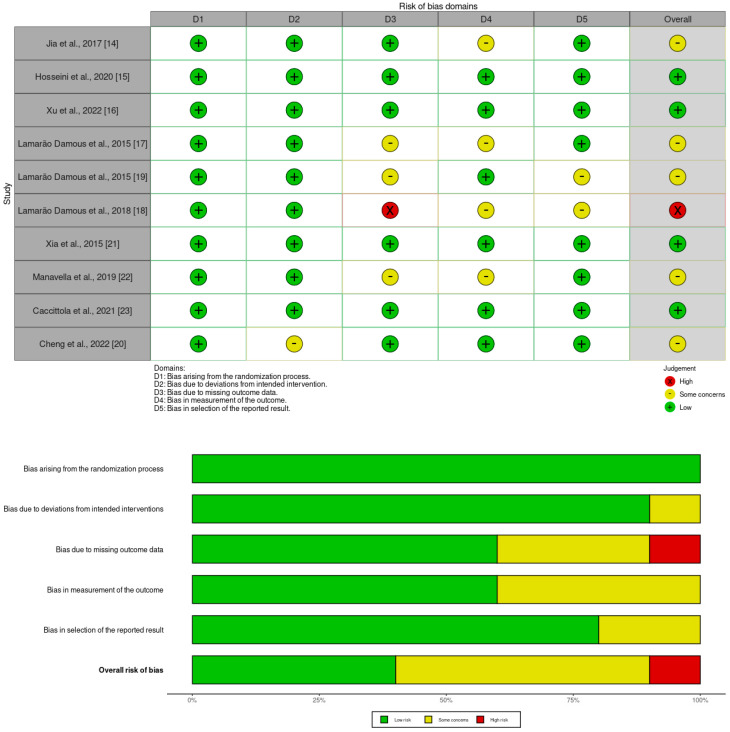
Summary of risk of bias in primary studies [14,15,16,17,18,19,20,21,22,23].

We considered the risk of bias using Version 2 of the Cochrane risk-of-bias tool for randomized trials (RoB 2). RoB 2 is structured into a fixed set of five domains of bias, focusing on different aspects of trial design, conduct, and reporting through which bias might be introduced into the result. The mentioned domains are listed in Figure 2. Within each domain, a series of signaling questions aims to elicit information about features of the trial that are relevant to the risk of bias. Response options for the signaling questions are: (1) Yes; (2) Probably yes; (3) Probably no; (4) No; (5) No information. A proposed judgment about the risk of bias arising from each domain was generated by an algorithm and based on answers to the signaling questions. RoB 2 is conceived hierarchically, and responses to signaling questions provide the basis for domain-level judgments about the risk of bias. In turn, these domain-level judgments provide the basis for an overall risk-of-bias judgment for the specific trial result being assessed. Judgment can be ‘Low’ or ‘High’ risk of bias, or it can express ‘Some concerns’. A summary of this assessment is provided in Figure 2. In terms of the overall risk of bias, one study is assessed as high risk [18], while there are some concerns about the overall risk of bias in the other five studies mainly arising from the risk of bias in the measurement of the outcome and bias due to missing outcome data [14,17,19,20,22].

## 4. Discussion

The latest clinical method of fertility preservation is based on the collection of ovarian cortical tissue fragments by multiple biopsies, their cryopreservation, thawing, and autotransplantation (whether to a heterotopic or orthotopic site). The first live birth after orthotopic ovarian tissue transplantation was reported in 2004 by Donnez and colleagues [38]. Since then, the improvements in technique have resulted in a live birth rate of 35–40%, with more than 200 live births being reported [39,40]. The American Society for Reproductive Medicine removed the experimental label from OTC procedures in 2019 [41]. However, as this fertility preservation modality is mainly intended for cancer patients, the risk of malignant cell contamination of the graft poses a severe limit in clinical practice. Alternative approaches are being investigated, such as decellularization techniques wherein individual ovarian follicles are being isolated and encapsulated in 3D bioscaffolds to form an artificially engineered ovary [42,43].

The success of the OTC procedure is dependent on the preservation of ovarian follicles. The first obstacle that the tissue faces is the freezing and thawing process, and the next obstacle, during which the majority (more than 50 percent) of follicles is being damaged, is the period of post-transplantation tissue hypoxia which persists until the establishment of adequate vascularization and tissue blood supply. Shift to the aerobic metabolism following neo-vascularization sets off the formation of oxygen radicals, which further induce tissue trauma [2]. A dual response is triggered when ovarian tissue is transplanted that involves follicle death but also the accelerated growth of primordial follicles to more advanced stages. Phenomenon of transplantation induced large-scale proliferation of quiescent follicles, known as the burnout effect, leads to uncontrolled growth and subsequent depletion of the ovarian reserve. It is caused by the triggering of signaling pathways involved in cell survival and proliferation. Indeed, the immediate post-transplantation period is characterized by hypoxia, and the cellular response to ischemia is bimodal, causing either cell death or activation of cell survival mechanisms, including the PI3K/Akt pathway. Increased expression of hypoxia inducible factor 1 activates the PI3K/Akt signaling pathway, resulting in cellular proliferation. Akt phosphorylation returns to normal values as graft angiogenesis progresses and normoxic conditions are restored [35]. The Hippo signaling pathway controls organ size by regulating cell proliferation, apoptosis, and stem cell self-renewal. It includes a number of negative growth regulators acting in a kinase cascade that eventually inactivate the key Hippo signaling effectors YAP/TAZ. When Hippo signaling is impaired by ovarian tissue fragmentation, nuclear levels of YAP are increased, leading to stimulation of cell growth and proliferation. The PI3K and Hippo pathways are interconnected, both contributing to the follicle burnout in the early post-transplantation period [44]. Ultrastructural alterations in the cytoplasmic organelles, cytoskeleton, and cell membrane are often observed in transplanted tissue oocytes [2]. Therefore, great efforts are being made to improve graft revascularization, which would enable better graft survival and improve the efficiency of the transplanted tissue [5,45].

The regulation of angiogenesis is a complex process that requires coordinated interaction of various angiogenic and antiapoptotic factors [46]. There are several strategies for minimizing post-transplantation ischemic graft damage, such as mechanical tissue injury, which promotes granulation tissue formation in the grafting site, the use of antioxidants (N-acetylcysteine in particular), as well as stimulation of angiogenesis by delivery of various factors and hormones such as VEGF111, VEGF165, FGF2, erythropoietin, angiopoietin-2 and sphingosine-1-phosphate. An additional option is co-transplantation of ovarian tissue with specific cell lines, such as exogenous endothelial cells or mesenchymal stem cells [8,47,48,49,50,51,52,53,54].

The concept of vascular bed-preparing and promoting vascularization of the planned grafting site before transplantation was investigated in studies by Manavella and Cacciotolla [22,23]. There were also reported attempts to prepare the bed with encapsulated angiogenic factors such as VEGF127 and stromal cells enriched in CD34 cells [55,56].

To the best of our knowledge, this systematic review is the first to compile data on the effect of stem cells on cryopreserved ovarian tissue grafts. Even though the studies did not exhibit the comparable data required for meta-analysis, they provided detailed information that was sufficient for a structured comparison of outcomes. Overall, the majority of the results support the fact that the addition of stem cells to the transplanted ovarian tissue leads to an accelerated process of angiogenesis, increased vessel density, a shortened period of tissue hypoxia and decreased levels of apoptosis, which consequently results in a superior follicle survival rate together with accelerated restoration of endocrine ovarian function.

Primordial follicles are maintained in a dormant state by various inhibitory molecules. Triggers that cause their recruitment are still insufficiently researched in humans. The early phase of follicle growth is gonadotrophin-independent and controlled by various autocrine and paracrine interactions. Once follicles reach antral stage, cyclic stimulation via gonadotrophins becomes necessary for their further recruitment and development. The establishment of axis stimulation and E2 production resulting from positive regulation of follicular growth presents a breakpoint in the resumption of oogenesis [57]. Moreover, paracrine factors play a key role in the process of selecting the dominant follicle by adjusting follicular response to gonadotrophins, oocyte maturation, completion of meiotic division, follicle rupture and luteinization in human cryopreserved ovarian tissue following xenografting [21,58,59]. Maintenance of the aerobic metabolism of oocytes in an ovarian tissue graft takes place at the expense of the low metabolic needs of resting primordial follicles and the development of granulosa cells in hypoxic conditions [23,60].

Alternative procedures for improvement of graft quality include cryopreservation of the whole ovary with vascular pedicle followed by anastomosis to an artery in the recipient site. Although more attractive than OTC in theory, whole organ manipulation is more demanding from surgical and cryobiological perspectives [61,62].

Adult stem cells are partially differentiated multipotent cells with high proliferative potential and the ability for multidirectional differentiation of their daughter cells (depending on cellular surroundings). They present the regenerative reserve of an organism. Easy accessibility, high proliferation capacity, low immunogenicity and plasticity make mesenchymal stem cells an ideal cell lineage for clinical usage [11].

Given the ethical dilemmas about research in the field of human fertility, such research is often conducted in vitro. Analyzing in vitro OTC cultures, it was observed that co-cultured mesenchymal stem cells secrete bioactive molecules with regenerative, antiapoptotic, and angiogenic effects into the culture medium. These include FGF, VEGF, IGF1, HGF, and angiopoietin-2, which act by stimulating Wnt/Beta-catenin and PI3K/AKT/mTOR signaling pathways that play an essential role in human angiogenesis [14,15,16]. Additionally, tissue ischemia induces the differentiation of MSCs into pericytes and endothelial cell-like lineages, which provide required cellular components to stabilize evolving graft neovascularization [21].

Lamarão Damous and colleagues in their in vivo experiments on Wistar rats investigated the option of direct injection of ASCs to the grafting site; however, this resulted in an exacerbated intrinsic inflammatory response with the consequent increase in cell apoptosis and ovarian tissue fibrosis [17]. In contrast, the delivery of ASCs to the grafting site through a gelatin-based sponge as an acellular scaffold did not induce an inflammatory reaction in grafted tissue. The acellular matrix enabled the slow and gradual release of ASCs to the ovarian graft surface [19]. The last step of their research was the cell-free therapy with the ASC secretome. Its application to frozen-thawed ovarian graft resulted in an increased inflammatory process and aggravation of ischemic injury damage [18].

Finally, the effect of SCs on human frozen-thawed ovarian tissue transplanted to immunodeficient mice was investigated. In contrast to the transient effect of delivery of bioactive molecules to the grafting site, co-transplanted stem cells persistently secreted various coordinated angiogenic factors which induced longer-lasting angiogenesis and provided an antiapoptotic effect [21].

Cheng et al. investigated the effect of hypoxia-preconditioned human umbilical MSCs on follicular survival. Preconditioning of stem cells in a hypoxic medium stimulates paracrine secretion of angiogenic factors and thereby shortens the post-grafting hypoxia period. It is also proven to suppress the expression of proinflammatory cytokines [20]. In addition to bioactive factor-secretion, the acquisition of CD34 and CD31 antigens on the ASC membrane proved their differentiation to endothelial-like cell lineage [22,23].

Still, there are many limitations to this type of research. Firstly, a direct assessment of grafted tissue oxygenation was not performed. All evaluated parameters are only indirect indicators of tissue oxygen levels. Furthermore, a long-term follow-up needs to be performed to exclude the possibly transient effect of the presented treatment. The main setback in the field of fertility research is the current impossibility of translation of presented modalities into clinical practice due to complex ethical dilemmas. Medical interventions in the gonadal tissue have an impact not only on the patient’s health condition but also on potential descendants. Detailed research in vitro and on animal models should precede the introduction of the procedure into clinical practice. Even in the case of applying this procedure to human patients, it would be challenging to compare the clinical results of OTC with the addition of stem cells considering the great number of variables that affect main outcome, i.e., live childbirth. We believe that, at this time, human stem cells and gonadal tissue transplanted to immunodeficient animal models present the most favorable form of research.

## 5. Conclusions

The achievements of modern medicine require intensive research and advancements in the fields of reproductive medicine and assisted reproductive technologies. Improvement in the viability of transplanted ovarian tissue would mean a great step forward in the field of oncofertility. The application of stem cells to the grafting site has been proven to support vascular promotion and thereby shorten the period of tissue hypoxia, which is reflected in the increased number of remaining viable follicles and faster recovery of ovarian endocrine function. Having said that, the presented results prove the complexity and unpredictability of the interplay of various paracrine factors and their influence on the preservation of transplanted tissue. Further research is needed before implementing the use of stem cells in OT cryopreservation and transplantation procedures in clinical practice.

## Data Availability

The data that support the findings of this study are available upon request from the corresponding author.

## Supplementary Materials

The following supporting information can be downloaded at: https://www.mdpi.com/article/10.3390/biology13050342/s1, Table S1: Data extraction form.
