# Supplementary material for: Enhancement of Vascularization and Ovarian Follicle Survival Using Stem Cells in Cryopreserved Ovarian Tissue Transplantation—A Systematic Review
Source: Biology (Basel). 2024 May 14;13(5):342. doi: 10.3390/biology13050342 (PMC11117700; doi:10.3390/biology13050342)
Supplement: Supplementary file 1 [file biology-13-00342-s001.zip › biology-2992296-supplementary.pdf]

Table S1. Data extraction form

**Characteristics of the study**	
	authors
	year of publication
**Characteristics of study design**	
	in vitro/in vivo
	experimental groups
	origin of ovarian tissue
	number and age of tissue donors
	method of ovarian tissue cryopreservation
	size of ovarian tissue fragments
	type and origin of stem cells
	cell line passage number
	ovarian tissue transplantation site
	recipient animal
	type of in vitro culture; culturing medium
**Outcomes**	
**Histological evaluation**	tissue preparation and staining
	survival of ovarian follicles in total and survival of different stages of ovarian follicles; follicle density
	tissue changes (e.g. fibrosis, hyalinization)
**Immunohistochemistry**	angiogenesis markers (e.g. CD34, CD31, VEGF)
	apoptosis markers (e.g. AC-3)
	cellular proliferation markers (e.g. Ki67)
	oxidative stress markers (e.g. HIF1a, Nrf2, 80HdG)
**TUNEL assay**	follicle apoptosis rate
**RT-PCR**	mRNA expression of genes coding for angiogenesis-related growth factors (e.g. VEGF, ANGPT2, IGF1, FGF2)
	mRNA expression of genes related to Wnt/β-catenin signaling pathway
	mRNA expression of genes related to oxidative stress
**Hormonal status**	concentration of hormones (E2, anti-Mullerian, progesterone, FSH)
	time of resumption of the menstrual cycle in rats/mice
